# Infective Endocarditis: Clinical Characteristics and Echocardiographic Findings

**DOI:** 10.3389/fcvm.2022.789624

**Published:** 2022-04-04

**Authors:** Hoorak Poorzand, Fatemeh Hamidi, Fereshte Sheybani, Fereshteh Ghaderi, Afsoon Fazlinezhad, Hedieh Alimi, Leila Bigdelu, Saeede Khosravi Bizhaem

**Affiliations:** ^1^Division of Cardiovascular Medicine, Cardiovascular Department, Vascular and Endovascular Surgery Research Center, Faculty of Medicine, Mashhad University of Medical Sciences, Mashhad, Iran; ^2^Cardiovascular Department, Faculty of Medicine, Mashhad University of Medical Sciences, Mashhad, Iran; ^3^Cardiovascular Diseases Research Center, Birjand University of Medical Sciences, Birjand, Iran; ^4^Department of Infectious Diseases and Tropical Medicine, Faculty of Medicine, Mashhad University of Medical Sciences, Mashhad, Iran

**Keywords:** infective endocarditis, congenital heart disease, mortality rate, follow-up care, echocardiography

## Abstract

**Purpose:**

Infective endocarditis (IE) remains a disease with high morbidity and mortality. The aim of this study was to determine the clinical characteristics and echocardiographic features of patients with IE.

**Methods:**

We analyzed patients with either definitive or probable diagnosis of IE who were hospitalized in a teaching hospital in Mashhad, Iran between June 2011 and January 2020. Patients who survived were followed up by echocardiography for at least 6-month after hospital discharge.

**Results:**

A total of 82 cases with IE were included of which 62 (75.6%) received definitive diagnosis. The mean age was 39.7 ± 18.7 years and 52 (63.4%) were male. The most common preexisting structural cardiac abnormality that predispose patients to IE were congenital heart diseases (28 %) of which bicuspid aortic valve was more common (*n* = 12, 14.6%), followed by ventricular septal defect (*n* = 9, 11%) and Tetralogy of Fallot (TOF) (*n* = 2, 2.4%). Three (3.6 %) cases had rheumatic heart disease and 12 (14.6 %) were injecting drug users. The most common causative pathogen was *Staphylococcus aureus*, detected in 7 (19.4%) cases. Follow-up echocardiography revealed right or left ventricular failure in 10 (12.1%) cases. Cardiac complications occurred in 41 (50%) cases and systemic complications in 63 (76.8%). All-cause mortality was 41.5% (*n* = 34) and 6 (18.1%) patients died due to cardiovascular complications.

**Conclusions:**

The short- and long-term prognosis in IE was poor and the predictors for in-hospital and 1-year mortality were defined as heart failure and septic shock. Congenital heart disease and intravenous illicit drug using (IVDU) were the most common predisposing condition which may necessitate a revision in the IE prophylaxis recommendations.

## Introduction

Infective endocarditis (IE) is a life threatening condition that is associated with a great economic burden (1). Despite the advancement in the medical treatments and surgical techniques, mortality and morbidity of IE remains high: one in five patients dies during the initial hospital admission (2). Furthermore, long term survival after IE is estimated <50% (3). Frequent delayed and missed diagnosis of IE can impact patients' chance of recovery and survival (4). The annual incidence of IE in high income countries reached up to 9 cases per 100,000 population and the incidence of hospitalization due to IE has almost doubled in the recent decades (5, 6). Although there is limited information regarding the incidence of IE in low- and middle-income countries, with substantial regional variations in the reported data, the overall incidence of IE appear to be higher, as compared to high-income countries (7).

Recently, a shift in the IE predisposing conditions has occurred in high-income countries from chronic rheumatic heart disease to intravenous illicit drug using (IVDU), degenerative valve disease, and intracardiac devices (3, 6, 8). Rheumatic and congenital heart diseases, however, are still the most important predisposing conditions of IE in the low- and middle income countries as well as Middle Eastern countries (9).

Serious cardiac and systemic complications are common in IE. Recent studies reported about 80% of IE patients experience at least one complication (3, 7). Of serious systemic and cardiac complications of IE, the most important factors affecting the clinical outcome are congestive heart failure, valvular dysfunction, and thromboembolic events. Congestive heart failure is the most common cardiac complication occurring in about 50–60% of the episodes of IE (1).

Although cross-sectional and cohort studies reported short and long-term survival rates of IE patients, there is limited information available about the long-term echocardiographic outcome of patients who survived from episodes of IE. Available studies on IE in Iran reported an increasing trend of hospitalization due to IE in the country, with 7–25% mortality rate (4, 10–12). Here, we describe the clinical and demographic characteristics, as well as echocardiographic features of hospitalized patients with IE in a large teaching hospital in Mashhad, Iran. We also analyzed the predisposing conditions of IE, in hospital and long-term mortality rates of patients and long-term cardiac outcome by follow-up echocardiography.

## Methods

**Figure 1:**
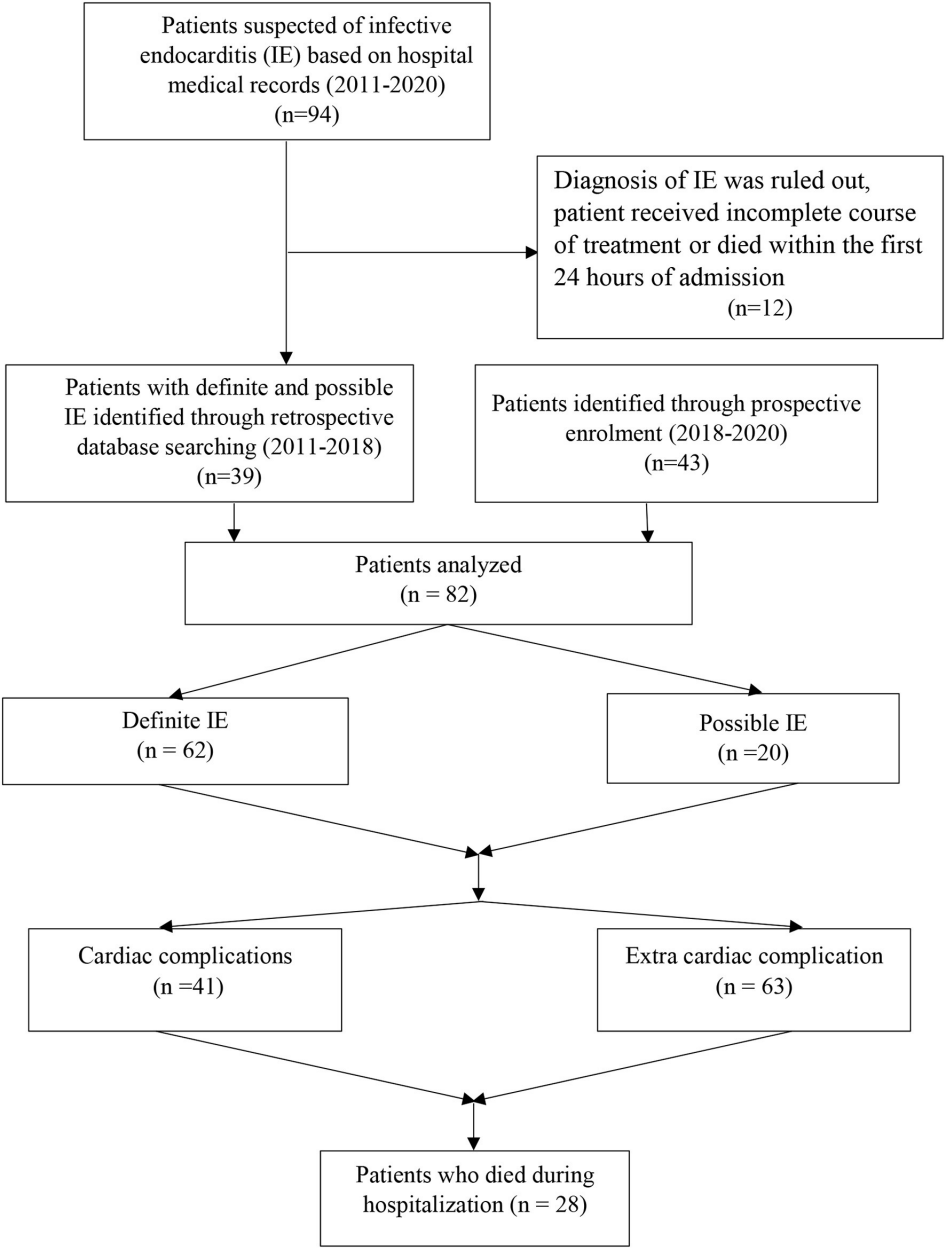
Enrolment protocol of patients with definite or possible infective endocarditis.

It was a retrospective cohort study that was conducted in a 1,000 bed teaching hospital affiliated to Mashhad University of Medical Sciences, Iran between May 2011 and January 2020 (Figure 1). We reviewed the medical records of all patients who were hospitalized with either definitive or probable diagnosis of IE, based on the modified Duke criteria of the European Society of Cardiology guideline 2015. Patients were excluded if the diagnosis of IE was ruled out, patient received incomplete course of treatment because of leaving the hospital before 7 days of hospitalization, or patient died within the first 24 h of admission. Data on patient history, implantable cardiac devices, previous IE, symptoms and signs on admission, laboratory findings, echocardiographic evaluations, treatment, complications, and outcome were retrospectively collected using patients' medical records.

Patients underwent echocardiography at the time of hospital admission for diagnosis and if needed, for evaluation of complications during admission, and once at least six months after discharge if survived at the time of follow up.

### Definitions

Major systemic embolic events (SEE) were described as occurrence of any major embolic event: peripheral limb ischemia, pulmonary embolization, neurologic accident and coronary ischemia.

Pericardial effusion was categorized to mild (<1 cm), moderate (1–2 cm) and Severe (>2 cm) by fluid thickness in echocardiography.

Indications of cardiac surgery were defined according to 2015 European society of cardiology guideline (13).

According to the 2013 American College of Cardiology and American Heart Association (ACCF/AHA) guidelines, severe heart failure was defined as LVEF <30% associated with NYHA III –IV (14).

### Statistical Analysis

Normality of data was assessed using the Kolmogorov-Smirnov test. Mean and standard deviation (SD) were used for normally distributed variables and median and interquartile range for non-normally distributed variables. Frequency and percentage were used for categorical variables. The Chi-square or Fisher's exact tests were used to compare categorical variables. Kaplan Meier curve and Cox proportional hazard model was done by STATA (version 14). The level of statistical significance was *p* < 0.05.

## Results

### Study Population

From May 2011 to January 2020, 94 patients were hospitalized with primary diagnosis of IE, of which 12 were excluded. Finally, 82 cases were analyzed with mean age of 39.7 ± 18.7 years, of whom 52 (63.4%) were male (Table 1). Elderly patients (≥65 years) accounted for 8 (9.7%). Twenty (21.6%) cases had possible and 62 (78.4%) definitive diagnosis of IE, according to modified Duke criteria.

**Table 1:** Patient characteristics and the underlying condition.

**Variable**			***N*** **(%)**
Age, (<65 years)			74 (90.3)
Gender (male)			52 (63.4)
Comorbidities			
		Chronic obstructive pulmonary disease (COPD/asthma)	17 (20.7%)
		Hypertension	14 (17%)
		End stage renal disease (ESRD)	13 (15.8%)
		Diabetes mellitus	13 (15.8)
		History of CAD	11 (13.4)
		Immunocompromised state	8 (9.7)
		Heart failure	7 (8.5)
		Cancer	3 (3.6)
Pregnancy			2 (2.4)
Duke criteria			
		2 major criteria	37 (45.1)
		1 major criteria	40 (48.7)
		3 minor criteria (with and without major criteria)	27 (32.9)
Predisposing condition			
		Congenital heart disease	23 (28)
		Bicuspid aortic valve	12 (14.6)
		VSD	9 (11)
		TOF	2 (2.4)
		Central venous catheter	16 (19.5)
		IVDU	12 (14.6)
		Prosthetic valve	8 (9.7)
		Bioprosthetic valve	1 (1.2)
		Mechanical valve	7 (8.5)
		Degenerative valve disease	6 (7.3)
		Rheumatic heart disease	3 (3.6)
		Device implantation	4 (4.9)
		Previous IE	1 (1.2)
Prior antibiotic therapy within the last 2 weeks			28 (34.1)
Recent dental procedure			2 (2.4)
New York Heart Association (NYHA) classification			
I			12 (14)
II			24 (29)
III			28 (34)
IV			19 (23)

The most common non-cardiac underlying comorbidity was chronic obstructive pulmonary disease (COPD/asthma) in 17 (20.7%) cases, followed by hypertension in 14 (17%), end stage renal disease (ESRD) requiring hemodialysis in 13 (15.8%), and diabetes mellitus in 13 (15.8%). Eight (9.7%) cases were taking immunosuppressive drugs, and 3 (3.6%) had active malignant neoplasms.

Sixty-four (89.0%) cases had native valve IE (NVE) and 8 (11.0%) prosthetic valve IE (PVE). Of those with NVE, 3(4.6%) and 6 (9.2%) had rheumatic and degenerative heart disease, respectively.

Congenital heart diseases were the most common predisposing condition (*n* = 23, 28 %), with bicuspid aortic valves (BAVs) (*n* = 12, 52.1%) and ventricular septal defect (VSD) as the predominant lesions (*n* = 9, 39.1%). The most common type of VSD in patients was peri membranous and none of them underwent surgical VSD closure. IE was associated with intravenous illicit drug using (IVDU) in 12 (14.6%) episodes and central-venous catheter infection in 8 (9.7%). Endocarditis involved cardiovascular implantable electronic device (CIEDs) in one patient with a history of permanent pace maker.

### Clinical and Laboratory Characteristics

The median duration of symptoms prior to hospital admission was 21 days [interquartile range (IQR), 26–75]. The most common symptoms were fever in 71 (86.5%), followed by malaise in 71 (86.5%), loss of appetite in 65 (79.2%), shortness of breath in 62 (75.6%), cough and excessive sweating, each in 31 (37.8%), dizziness 25 (30.4%), muscle pain 25 (30.4%), nausea/vomiting 20 (24.3%), chest pain 15 (18.2%), arthralgia 12 (14.6%), headache 11 (13.4%), and syncope 4 (4.9%) (Table 2). On admission, cardiac murmur was the most frequent sign (*n* = 71, 86.5%), and major embolic events (*n* = 31, 37.8%) and congestive heart failure (*n* = 19, 23.1%) were the most common complications that were identified during physical examination and initial diagnostic workup (Table 2).

**Table 2:** Clinical characteristics, laboratory features, with systemic and cardiac complications on admission.

**Variables**			***N*** **(%)^†^**
Symptoms	Malaise		71 (86.5)
	Fever		71 (86.5)
	Loss of appetite		65 (79.25)
	Shortness of breath		62 (75.6)
	Cough		31 (37.8)
	Sweating		31 (37.8)
	Dizziness		25 (30.4)
	Muscle pain		25 (30.4)
Signs	Cardiac murmur		71 (86.5)
	Congestive heart failure (NYHA IV)		19 (23.1)
	Peripheral stigmata	Janeway lesions	12 (14.6)
		Osler's node	2 (2.4)
		Roth spots	1 (1.2)
	Clubbing		1 (1.2)
Cardiac and systemic complications	Major embolic events		31 (37.8)
	Limb ischemia		1 (1.2)
	Cerebrovascular complications	TIA	1 (1.2)
		Ischemic stroke	14 (17.0)
		Hemorrhagic stroke	2 (2.4)
	Pulmonary emboli		12 (14.6)
	Septic shock		8 (9.7)
	Acute pulmonary edema		7 (8.5)
	Cardiogenic shock		4 (4.9)
	Coronary artery involvement (Left circumflex and right coronary artery)		2 (2.4)
Causative pathogen	Positive result		36 (43.9)
	*Staphylococcus aureus*		7 (19.4)
	MERSA (methicillin-resistance *Staphylococcus aureus*)		3(8.3)
	Viridans streptococci		3 (8.3)
	Enterococcal species		3 (8.3)
	Pseudomonas species		3 (8.3)
	HACECK		2 (2.4)
	Candida		2 (2.4)
	Acinetobacter		1 (1.2)
	Brucellosis (Positive tissue sample culture)		1 (1.2)
	Others (staph coagulase positive, staph epidermis, strep group D, strep agalactiae, kelebsiella pneumonia, …)		14 (17.07)
	BCNIE		46 (56.1)
Other laboratory findings	Parameter	Median	IQR (Q3, Q1)
	Hemoglobin (mg/dl)	9.2	2.9 (11, 8.1)
	WBC (×10^3^)	8.9	7.4 (13.2, 6.8)
	Platelets (×10^3^)	166	196.5 (304, 107.5)
	Creatinine, mg/dl	1.1	1.3 (2.2, 1.9)
	CRP	90	44.5 (116, 74.5)
	ESR	69	53.5 (92, 38.5)

† *Median and interquartile range were used. BCNE, Blood culture negative endocarditis*.

Involvement of the coronary arteries occurred in three patients: the first was a patient who developed left circumflex (LCX) artery embolism with aneurysmal formation in the proximal part detected by coronary angiography. The second case was a 61-year-old diabetic man who presented with fever and arthritis of hip joint. He developed anterior ST-elevation myocardial infarction (STEMI) caused by left coronary artery embolism during hospitalization. On echocardiography, he had a large mobile vegetation on the mitral valve. Last patient was a 50-year-old woman who developed a rare complication of pseudo aneurysm and extensive abscess formation in the anterior wall of the aortic root and right coronary artery during hospitalization.

The causative pathogen was isolated from blood culture in 36 (43.9%) cases. The most common pathogen was *Staphylococcus aureus* (*n* = 7, 19.4 %) of which 3 (42.8%) were methicillin resistant, followed by viridans s*treptococci*, enterococcal species, and pseudomonas species (3, 8.3% each). Acinetobacter was detected in one patient. Forty-six (56.1%) cases were categorized as blood culture negative infective endocarditis (BCNIE) (Table 2).

### Echocardiographic Features on Admission

All patients underwent trans-thoracic echocardiography (TTE), and in 43 (54.4%) cases trans-esophageal echocardiography (TOE) was also done (Table 3).

**Table 3:** Echocardiographic characteristics.

**Variable**				***N*** **(%)**
Vegetation location	Single valve involvement	Aortic	Native	13 (15.9)
			Biologic	1 (1.2)
		MV	Native	16 (19.5)
			Mechanical	4 (4.9)
		TV	Native	15 (18.3)
	Multivalve involvement	Ao V + MV	Native	9 (10.1)
		Ao V + MV	Mechanical	3 (3.6)
		MV+ TV	Native	2 (2.4)
	Ao V (native) and IVS (VSD case)			3 (3.6)
	TV (native) and IVS (VSD case)			2 (2.4)
	MV (native) and SVC			4 (4.9)
	Isolated SVC			4 (4.9)
	Isolated IVS (VSD peri-membranous)			3 (3.6)
	LTGA(VSD)			1 (1.2)
	Pulmonary conduit (TOF)			1 (1.2)
	RV wall (TOF)			1 (1.2)
	Total			82 (100)
	TV		Hx of IVDU	11 (57.8)
			No Hx of IVDU	8 (42.2)
Complicated IE	Ao V			52 (63.2)
	MV			52 (63.1)
	TV			31 (37.8)

*AoV, Aortic valve; MV, Mitral valve; TV, Tricuspid valve*.

In 17 (26.6%) of 64 cases with NVE, the infection involved right-sided valves, in 45 (70.3%) left-sided valves, and in 2 (3.1%) there was bilateral valve involvement. The most common site of vegetation was mitral valve in 24 (29.2%) episodes, followed by tricuspid valve in 17 (20.7%) and aortic valve in 17 (20.7%). Fourteen (17%) cases had vegetation at multiple sites (multivalve involvement). The median diameter of vegetations on aortic, mitral and tricuspid valves were 10, 11.5, and 14 mm, respectively.

In those patients with right-sided valve vegetation, 11 (57.8%) cases had history of intravenous drug use, 2 (10.5%) had cancer and receiving chemotherapy *via* venous port, and 2 (10.5%) were on hemodialysis. One patient was a healthy woman with a history of eyebrow tattooing 2 weeks prior admission.

The most common type of valve involvement was regurgitation in 66 (80.4%) cases and the most common valve complication was perforation in 41 (50%): aortic valve in 17 (20.7%) cases, and mitral and tricuspid valve, each in 12 (14.6%).

The median of ejection fraction (EF) and median of pulmonary arterial systolic pressure (PAP) were 47.5% (IQR, 55–50) and 47.5 mm Hg (IQR, 45–30), respectively. Left ventricular enlargement and right ventricular dysfunction were identified in 19 (24.1%) and 36 (45.6%) cases, respectively. Although echocardiographic evidence of severe pericardial effusion and tamponade were not detected in any patients, 31 (39.2%) episodes were complicated with mild or moderate pericardial effusion.

### Outcome

The mean length of hospital stay was 35 (IQR, 45–18.75) days. Seven (8.5%) cases transferred to the intensive care unit (ICU) with median length of ICU stay of 6 days (IQR, 5-0.5).

The most common complication during hospital stay was adverse drug reactions related to antibiotic therapy that were occurred in 27 (32.9%) cases, followed by acute renal failure in 19 (23.2%), and congestive heart failure in 14 (17.1%) (Table 4). Five (6.1%) cases experienced cardiogenic shock, twenty-two (26.8%) had persistent fever after seven days of antibiotics therapy and 7 (8.5%) had persistent bacteremia after 48 h of antibiotic therapy. New embolic events occurred in 10 (12.2%) cases during hospital admission. Among those who experienced adverse drug reactions related to antibiotics, the most frequent complication was acute kidney injury which was attribute to aminoglycoside and/or glycopeptide classes of antimicrobials.

**Table 4:** Frequency of systemic and cardiac complications during hospital admission.

**Variables**	***N*** **(%)**
Adverse drug reactions related to antibiotic therapy	27 (32.9)
Acute renal failure	19 (23.2)
Congestive heart failure	14 (17.07)
Glomerulonephritis	8 (9.8)
Neurologic complications	8 (9.8)
Stroke	3 (3.7)
Cerebral mycotic aneurysm	3 (3.7)
Intracerebral hemorrhage	2 (2.4)
Septic shock	7 (8.5)
Splenic abscess	1 (1.2)
Coronary artery embolism	1 (1.2)
New abscess formation in aortic valve	1 (1.2)

Cardiac surgery was indicated in 59 (72%) cases, of which 34 (57.6%) underwent heart surgery. The most common indication of cardiac surgery was severe heart failure in 46 (78%). Early post cardiac surgery death occurred in 7 (8.5%) episodes during hospital stay.

In hospital mortality occurred in 28 (34.1%) cases. Death was attributed to non-cardiac causes in 21 (77.7%) episodes and cardiac complications in 6 (22.3%). Non-cardiac causes of death included septic shock and multi organ failure in 13 (15.8%), neurologic catastrophes in 4 (4.8%), neoplasia in 3 (3.6%), and pulmonary emboli in one case (1.2%).

### Follow Up

Using telephone follow up, patients who survived to hospital discharge were asked to return to the hospital for a clinical evaluation and follow up echocardiography. The median follow-up was 330 days (IQR, 1095-187.5). Follow up echocardiography was performed using TTE in 36 (3.9%) and TEE in 4 (4.9%) cases.

The median left ventricular ejection fraction (LVEF) was 53% (IQR, 55-50) on follow up echocardiography. Ten (12.1%) cases had persistent ventricular failure, including 2 patients with severe RV failure as complication of right-sided IE. One of them underwent redo-tricuspid valve replacement (redo-TVR) due to severe transvalvular regurgitation of repaired infected tricuspid valve. Persistent vegetation was noted in 2 patients after corrective heart surgery. Prosthetic valve malfunction including increased trans-valvular gradient, and pannus or thrombus formation were found in 5 cases.

Thirty-Nine (47.5%) cases responded the follow up call, of which 36 (43.9%) underwent follow up echocardiography. Nine (11%) cases lost to follow up and six (7.3%) patients died within 6 months after discharge.

One patient experienced recurrence of IE. He was 44-year-old intravenous drug users with methicillin-resistant staphylococcal right-sided IE that readmitted 3 months after the first episode of IE with cardiogenic shock and large obstructive vegetation on the TV leaflets resulting in severe stenosis and regurgitation. He died before surgical intervention could be performed.

Post-discharge complications identified in 18 (45%) patients, including heart failure in 6 (16.6%), healthcare-associated infections in 4 (11.1%), and renal failure in 3 (7.5%). Four (10%) cases underwent post-discharge cardiac surgery.

### Analytical Results

In cases with blood culture negative infective endocarditis (BCNIE), the frequency of congestive heart failure (i.e., NYHA functional class of III-IV) was higher than those with positive blood culture results (*n* = 40, 88.9% and *n* = 5, 11.1%, respectively) (p: 0.692). Furthermore, mortality rate was higher in the episodes of BCNIE in comparison to other cases (*n* = 28, 87.5% vs. *n* = 4, 12.5%) (p: 0.432).

Independent predictors of death (in-hospital and 1-year) on multivariate Cox proportional hazard model were heart failure (HR:2.299, 95% CI:1.053–5.021, *p* = 0.037) and septic shock (HR:14.304, 95% CI:6.183–33.090, *p* < 0.001) (Table 5).

**Table 5:** Univariate Cox proportional hazard model.

**Variables**	**HR (95% CI)**	***P*** **-value**
Renal failure (ESRD)	1.425 (0.620–3.276)	0.404
Immunosuppressive treatment	1.502 (0.526–4.292)	0.447
Septic shock	13.608 (6.009–30.817)	<0.001
Heart failure	2.210 (1.022–4.779)	0.044
Not performed surgery	1.571 (0.756–3.262)	0.226
Negative blood culture infective endocarditis (NBCIE)	0.801 (0.401–1.603)	0.531

**Figure 2:**
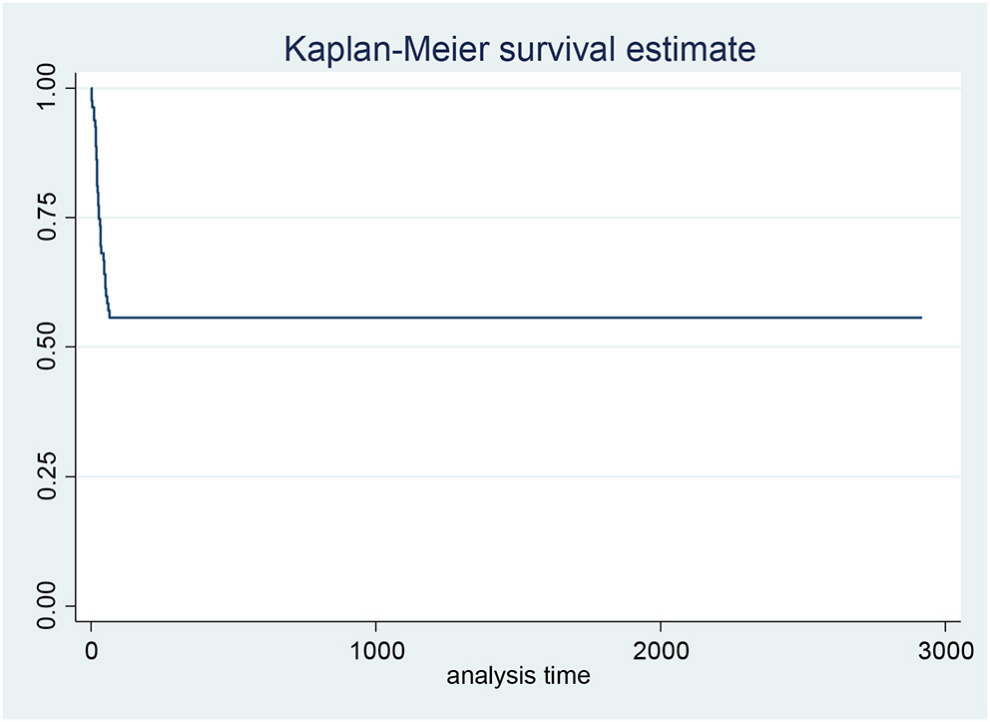
Kaplan-Meier curve showing the probability of mortality respect to the time of admission.

Kaplan Meier plot was used to assess the general pattern of survival which showed high intensity of mortality in initial days and then plateau to the end of study (Figure 2). The result of Kaplan Meier showed that the survival rate in the first 10 days was 0.94, in the first month was 0.7 and in 1 year was 0.56.

## Discussion

The short- and long-term prognosis in infective endocarditis (IE) was defined to be poor in this study. One third of IE cases died during hospital stay and 11% of those who survived to hospital discharge subsequently succumbed to death. Echocardiography revealed persistent ventricular failure in 12% of IE cases who were alive at median 11-month follow up. Previous research also indicates that 1-year mortality of IE has not changed over the last 2 decades, ranging from 15 to 30% (15). Poor outcome, despite the continues advancement in diagnostic and therapeutic modalities in the recent decades, can be partly related to delay in diagnosis of IE and hospitalization (4). High rates of systemic and cardiac complications on admission that were identified in our study deserve special attention and it could be due to late presentation and diagnosis. Of these, major embolic events and congestive heart failure were the most common ones on admission, occurring in 38 and 23%, respectively. More than half of these cases were classified as NIHA class III or IV on presentation. Higher NYHA classes in IE patients have been associated with increased morbidity and mortality (16). In the International Collaboration on Endocarditis-Prospective Cohort Study on 4,166 patients with definite IE (2000–2006), the presence of heart failure in IE patients was associated with significantly higher in-hospital mortality as compared to those without heart failure (29.7 vs. 13.1%, respectively, *P* < 0.001) (17).

The most common foci for major embolic events were central nervous system (CNS) and pulmonary circulation. Overall, IE was complicated by neurovascular complications in 30% of the episodes in our study. Similarly, rates of 20–40% have been reported for neurological complications in IE previously (5). Once neurological damage occurs, the mortality of IE increases up to 50% as compared to 21% in IE patients without neurological complications (18).

On multivariate analysis, the predictors of in-hospital and 1-year after discharge mortality were heart failure (HR:2.299, 95% CI:1.053–5.021, *p* = 0.037) and septic shock (HR:14.304, 95% CI:6.183–33.090, *p* < 0.001) (Table 5). SOFA score which is calculated the day of surgery was also identified as an independent associated factor with long-term mortality in a prospective study on 198 IE patients across 33 adult ICUs in France (19). Furthermore, heart failure within 3 months of admission, and collagen vascular diseases and alcohol abuse as underlying conditions were found as significant predictors for long-term mortality in another study on 326 episodes of IE in a Finnish teaching hospital (20).

Congenital heart disease and intravenous illicit drug using (IVDU) were the most common predisposing conditions of IE in our study. Chronic rheumatic heart disease, however, was responsible for <5% of cases of IE. This could be indicative to a shift toward non-rheumatic heart diseases as predisposing conditions of IE in Iran that is similar to the transition that occurred in most of developed nations (6). Chronic rheumatic heart diseases have been reported as the main underlying condition that predispose individuals to IE in developing countries (9). In high income countries, on the other hand, IE occur more commonly in the elderly and those with degenerative valvopathy, prosthetic valves, and cardiovascular implantable electronic devices (21). Another study from Tehran, Iran also described 602 cases of IE, of whom only 8.4% had rheumatic heart diseases as the underlying cardiac condition for IE (22). Nevertheless, the role of congenital heart disease as predisposing conditions of IE remains still significant in Iran. Of congenital heart disorders (CHD), bicuspid aortic valve (BAV) and ventricular septal defect (VSD) were the most commonly identified defects and Tetralogy of Fallot (TOF) accounted for <10% of cases. In a multicenter prospective observational cohort (2008–2016) of 736 patients with CHD in United Kingdom and Ireland, among 800 episodes of IE, the most common defect was TOF, followed by VSD and bicuspid aortic valve (22.8, 19.6, and 10.6%, respectively) (23). The significant role of these congenital heart diseases as predisposing conditions of IE probably necessitates a revision in the IE prophylaxis recommendations.

The most common affected heart valves were mitral and tricuspid valves in 29.2% and 20.7% of cases, respectively. We did not perform a trend analysis in this regard. However, a recent shift toward more frequent involvement of aortic and tricuspid valves in IE patients was reported in a 16-year study of IE patients in Tehran, Iran (11). High proportion of tricuspid valve involvement can be partly attributed to the significant proportion of IVDUs in our study. Nevertheless, about one third of cases with right-sided IE were not IVDU and their underlying conditions were cancer and being on hemodialysis. Right-sided IE is more often associated with IVDU, intra-cardiac devices, and central venous catheters, all of which become more prevalent over the past 20 years (24). Of these, IVDU is the most common predisposition for right-sided IE (24), as identified in our study.

In about half of IE patients in our study the causative pathogen was not isolated from blood specimens. This is higher than the rates reported by more developed countries that is about 10–20% (16, 25, 26). In those with culture-proven IE, *S. aureus* was the most common causative pathogen that was isolated in 19% of cases, followed by viridans *streptococci*, enterococci and pseudomonas species. A high proportion of blood culture negative IE (BCNIE) in our study can be attributable to several factors, including high rate of prior antibiotic therapy, inappropriate timing of specimen collection, and limited microbiology resources. BCNIE has been associated with difficulty in diagnosis and treatment, and poor outcome (26) and also in our study, it was associated with increased risk of heart failure, higher NYHA functional class, and higher risk of death. Antibiotic therapy within the 2 weeks before blood sampling was identified in about one third of IE cases and about 40% of those with BCNIE in our study. Furthermore, endemicity of some fastidious microorganisms such as Brucella species (27) or Q fever (28, 29) in Iran as the causative pathogens of IE can also be responsible for low yield of culture-based diagnostic tests. For example, in a study on 52 cases of BCNIE in Tehran, Iran, 31% of episodes were caused by *Coxiella burnetii* (29).

We encountered high rate of adverse drug reactions associated with antibiotic use, which was the most common complication during hospital admission for IE, and it could be at least partly explained by the lack of definite microbiological test results in a substantial proportion of our patients in order to de-Escalate the empirical antimicrobial regimen.

## Conclusion

The findings of our study showed that short- and long-term prognosis in infective endocarditis (IE) was poor, with more than one third IE cases die during hospital stay and one in ten of those who survived to discharge die within 6 months after discharge. Furthermore, persistent ventricular failure was detectable in 12% of IE cases who were alive at median 11-month follow up. Predictors of in-hospital and 1-year mortality were heart failure and septic shock. High rate of systemic and cardiac complications occurs in IE patients, with major embolic events and congestive heart failure were the most common complications on admission.

Congenital heart disease and intravenous illicit drug using (IVDU) were the most common predisposing conditions of IE in our study while chronic rheumatic heart disease was responsible for <5% of cases of IE; the finding that highlights a shifting from rheumatic heart disease as underlying abnormality toward a congenital heart disease, followed by IVDU and prosthetic heart valves. The important role of congenital heart diseases as predisposing conditions of IE may necessitate a revision in the IE prophylaxis recommendations.

A high proportion of blood culture negative IE (BCNIE) in our study can be attributed to high rate of prior antibiotic therapy, inappropriate timing of specimen collection and limited microbiology resources, as well as endemicity of some fastidious microorganisms such as Brucella species or Q fever as the causative pathogens of IE in Iran.

## Data Availability Statement

The original contributions presented in the study are included in the article/supplementary material, further inquiries can be directed to the corresponding author.

## Ethics Statement

The patients/participants provided their written informed consent to participate in this study.

## Author Contributions

HP and FH: study concept and design. FS and SK: analysis and interpretation of the data. FH, FG, AF, HA, and LB: drafting of the manuscript. All authors contributed to the article and approved the submitted version.

## Funding

This research was conducted with funding support from the vice-chancellery for research of Mashhad University of Medical Sciences (Research Project Number 971829).

## Conflict of Interest

The authors declare that the research was conducted in the absence of any commercial or financial relationships that could be construed as a potential conflict of interest.

## Publisher's Note

All claims expressed in this article are solely those of the authors and do not necessarily represent those of their affiliated organizations, or those of the publisher, the editors and the reviewers. Any product that may be evaluated in this article, or claim that may be made by its manufacturer, is not guaranteed or endorsed by the publisher.
